# Optimization of Rotary Friction Welding Parameters Through AI-Augmented Digital Twin Systems

**DOI:** 10.3390/ma18091923

**Published:** 2025-04-24

**Authors:** Piotr Lacki, Janina Adamus, Kuba Lachs, Wiktor Lacki

**Affiliations:** 1 Faculty of Civil Engineering, Częstochowa University of Technology, J.H. Dąbrowskiego 69 Str., 42-201 Częstochowa, Poland; janina.adamus@pcz.pl; 2 Faculty of Computer Science and Artificial Intelligence, Czestochowa University of Technology, J.H. Dabrowskiego 69 Str., 42-201 Czestochowa, Poland; kublac24@gmail.com; 3 Faculty of Mechanical Engineering, Czestochowa University of Technology, J.H. Dabrowskiego 69 Str., 42-201 Czestochowa, Poland; wiktor.lacki07@gmail.com

**Keywords:** Rotary Friction Welding, Artificial Neural Networks, digital twin, Finite Element Method

## Abstract

In this study, Artificial Neural Networks (ANN) were employed to develop a Digital Twin (DT) of the Rotary Friction Welding (RFW) process. The neural network models were trained to predict the peak temperature generated during the welding process of dissimilar Ti Grade 2/AA 5005 joints over a temperature range of 20–640 °C. This prediction was based on a parametric numerical model of the RFW process constructed using the Finite Element Method (FEM) within the ADINA System software. Numerical simulations enabled a detailed analysis of the temperature distribution within the weldment. Accurate temperature predictions are essential for assessing the mechanical properties and microstructural integrity of the welded materials. Artificial Intelligence (AI) models, trained on historical data and real-time inputs, dynamically adjust critical process parameters—such as rotational speed, axial force, and friction time—to maintain optimal weld quality. A key advantage of employing AI-augmented DT systems in the RFW process is the ability to conduct real-time (less than 0.1 s) optimization and adaptive control. By integrating a Genetic Algorithm (GA) with the DT algorithm of the RFW process, the authors developed an effective tool for analyzing parameters such as axial force and rotational speed, in order to determine the optimal welding conditions, which translates into improved joint quality, minimized defects, and maximized process efficiency.

## 1. Introduction

Joining different materials using conventional welding methods causes many problems due to their varying thermophysical properties and the formation of numerous intermetallic compounds, which impart brittleness to the welded joints. Additionally, most welding processes are affected by defects (such as porosity, shrinkage, hot cracking, and segregation), which form during solidification and reduce the quality of the joint [1]. Classical welding processes also pose physical hazards (excessive noise and dangerous thermal and optical radiation) as well as dust and gas pollution, which contains numerous hazardous substances that can cause air pollution and negatively impact human health and ecosystems.

To overcome the difficulties encountered in conventional welding, RFW, a solid-state joining process, can be used. In the RFW process, the heat required to join materials comes from the direct conversion of mechanical energy into thermal energy, resulting from friction in the contact area of the welded parts. This process avoids the melting of materials, thereby preventing solidification-related defects. Additionally, the lower temperature in the welding zone limits the formation of brittle intermetallic compounds, enabling the joining of different materials with varying physical properties that cannot be joined using conventional welding methods.

Therefore, RFW is widely used in the aerospace, automotive, oil and gas, and medical industries, where it joins parts of various shapes and sizes, such as turbine shafts, automotive parts, electrical connectors, cutting tools, and medical implants. This process allows the joining of similar [2] and dissimilar materials, such as AA7075 and AA2024 aluminum alloys [3], or aluminum and titanium alloys [4], enabling the creation of lightweight and durable structures. The RFW process is characterized by high weld quality, often comparable to or better than the strength of the base material.

With the development of AI and digitalization in production, new perspectives for creating products with predictable properties are emerging. The rapid development of AI tools, including Genetic Algorithms (GAs), Artificial Neural Networks (ANN), and other Machine Learning (ML) techniques, offers new possibilities for precise control of the RFW process. Digital Twin (DT) technology enables comprehensive data recording and analysis of welding process states, as well as full visualization of the process. These systems integrate ML algorithms with virtual replicas of the physical welding setup to predict and adjust key process variables such as rotational speed, axial pressure, and friction time, enhancing joint quality and mechanical properties. Advanced models like ANN and GA are used to analyze big datasets, uncover complex relationships between input parameters and weld outcomes, and propose optimal settings without the need for extensive physical trials. As stated in [5], AI techniques such as ANN and GA have been successfully applied to optimize welding parameters, achieving an accuracy level of 85–99% in predicting outcomes. Hybrid methods combining AI with traditional optimization strategies, like Response Surface Methodology (RSM), have been shown to enhance prediction accuracy and optimize process parameters effectively.

Simulation software, such as ANSYS 2024 R2 and SIMULINK 24.2 (part of R2024b), allow the analysis of material behavior under various welding conditions, enabling the identification of optimal parameters like friction pressure and rotational speed [6]. The use of DT systems facilitates real-time monitoring and adjustment of welding parameters, further improving the quality and efficiency of the welding process [5]. Studies highlight how these DT systems improve efficiency by minimizing defects and reducing material waste while enabling adaptive control in response to varying material behaviors. This approach not only accelerates product development cycles but also ensures greater consistency and performance in critical applications like aerospace and automotive manufacturing.

Although there are more and more successful implementations in research and industrial work, some authors [7] point out that a full evaluation of these solutions is impossible due to the lack of detailed, publicly available information. Additionally, the issue of defining a DT also remains unresolved [8], even though the DT concept has been proposed for over 20 years and is one of the main ideas associated with Industry 4.0. These challenges highlight the ongoing complexity and evolving nature of DT technology. In [8], the definitions of the DT concept in scientific literature were analyzed, tracing its origins in aviation and space to its latest applications in production and smart manufacturing.

DT offers immense potential in various domains of the product engineering process. As noted by the authors of paper [9], although current approaches for utilizing DT focus on separate disciplines, it is anticipated that the holistic use of DT models in product development and production will dominate future generations of products, enabling the competitive creation of high-performance products. The authors of [10] note that the stringent requirements for durability, reliability, and efficiency, especially for future aircraft and spacecraft, will need a change in the approach to their design, testing, and maintenance. The current approach—relying on statistical material properties, heuristic design principles, physical tests, and assumed similarity between test and operational conditions—is unlikely to meet extreme requirements.

The introduction of DT will integrate high-fidelity simulations with the onboard vehicle health management system, maintenance history, and all available data to accurately reflect the lifespan of its flying twin, ensuring an unprecedented level of safety and reliability. A similar transformation is occurring in the construction industry. While Building Information Modeling (BIM) provides procedures, technologies, and data for the standardized representation of building components and systems, the concept of DT offers a more holistic, socio-technical, and process-oriented characterization of complex artefacts. It utilizes the synchronization of cyber-physical bidirectional data flows, as noted in [11], paving the way for the concept of construction DT.

Paper [12] discusses how the DT algorithm enhances intelligent automation in the automotive, aerospace, construction and real estate industries. This paper outlines the concept, traces the evolution and development of DT, reviews key enabling technologies, examines trends and challenges, and explores its applications. While the integration of AI in RFW optimization has shown promising results, challenges remain in standardizing these techniques to different materials and welding scenarios. Further research is needed to fully explore AI’s potential in improving the adaptability and precision of welding processes.

## 2. Materials and Methods

This study serves as an extension and continuation of the work presented in [1,13], focusing on the prediction and optimization of RFW parameters. The numerical calculations conducted in this research were performed using the ADINA 24 Bentley Systems, Incorporated; Exton, PA, USA which facilitates the analysis of heat generation due to friction within an axisymmetric thermomechanical model.

Frictional resistance was modeled using a temperature-dependent friction coefficient to accurately represent material behavior during the welding process. The adopted thermomechanical model required precise determination of the thermo-elastic–plastic properties of the analyzed materials as a function of temperature. Friction welding simulations were conducted for varying welding parameters and time durations to assess their effects on the process. The numerical simulations enabled the prediction of temperature distribution within the weld cross-section, which is a critical factor for subsequent analyses. The resulting temperature fields provide essential data for evaluating structural transformations, hardness variations, residual stress, and material deformation. In this study, dissimilar joints of Ti Grade 2 and AA 5005 aluminum alloy, previously described in detail in [1], were utilized to construct the DTs models. AA 5005 belongs to the 5000 series aluminum alloys, where magnesium serves as the primary alloying element. This alloy is widely used as a construction material due to its relatively high strength, excellent formability, especially in its annealed state, superior fatigue resistance, good weldability, and robust corrosion resistance. Commercially pure titanium Grade 2 is known for its excellent corrosion resistance, relatively good formability, and moderate mechanical strength. These properties make it a suitable candidate for various industrial applications, especially when paired with lightweight aluminum alloys in dissimilar metal joining processes. The integration of numerical modeling and DT technology in this work contributes to a deeper understanding of the thermal and mechanical behaviors during RFW, leading to the optimization of welding parameters.

**Figure 1:**
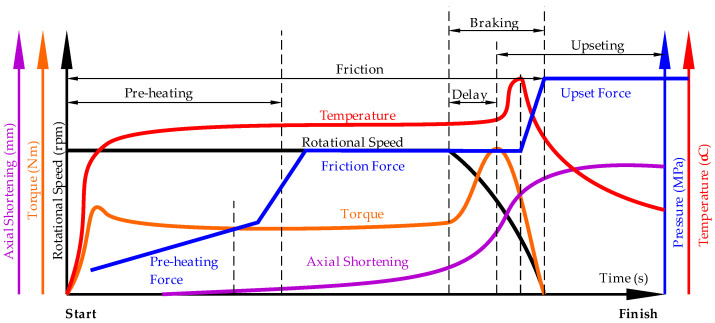
RFW process parameters considered in the DT framework.

The effectiveness of the DT depends on incorporating as many RFW process parameters as possible into the model. Figure 1 shows the key parameters of the RFW process included in the DT.

The maximum temperature at the faying surfaces during the RFW process is influenced by several key process parameters such as: rotational speed, axial force, friction coefficient and friction time. These factors determine the frictional heat generated, the efficiency of heat transfer, and the thermal and mechanical behaviors of the materials being joined. Previous studies [1,13] indicate that higher rotational speeds increase frictional heat generation due to the greater rate of sliding contact, leading to higher maximum temperatures at the interface. The maximum temperature generally rises with rotational speed, though an optimal range exists depending on the material properties. Higher axial forces increase frictional heat generation by enhancing frictional resistance. However, excessive axial force may reduce the temperature by accelerating material deformation and shortening the frictional sliding phase. While the maximum temperature initially rises with axial force, it can decrease at very high forces due to excessive material flow. Friction time determines how long heat is generated at the interface. A longer friction time allows the temperature to gradually increase, reaching higher maximum values. The maximum temperature increases with friction time until the system reaches thermal equilibrium.

The interaction of these parameters is often nonlinear, and the optimal settings depend on factors such as the material pair, component geometry, and the desired joint quality. High rotational speed, combined with moderate axial force and friction time, is generally optimal for achieving sufficient heat and plastic deformation. However, excessive axial force or excessively long friction times may lead to material defects, such as voids or cracking, caused by overheating or improper material flow. In this study, numerical models and simulations, including those based on the Finite Element Method (FEM), were employed to investigate the combined effects of these parameters. Furthermore, the use of AI-augmented Digital Twin (DT) systems enhances the ability to predict and optimize maximum temperatures and welding outcomes across varying conditions.

### 2.1. DT Based on MES

The Finite Element Method (FEM) plays a crucial role in the creation and functionality of Digital Twin (DT) systems, particularly in the case of complex physical systems and structures. FEM enables the simulation and analysis of how physical objects respond to various forces, stresses, and environmental conditions, thereby enhancing the accuracy and predictive capabilities of DT. FEM also enables stress, fatigue, and vibration analysis, allowing DT to predict potential failures. This predictive capability supports proactive maintenance strategies, reducing downtime and extending equipment life. Advanced FEM models, integrated with real-time sensor data, enable DT to adjust simulations based on current operating conditions. This dynamic updating improves performance predictions and decision-making. FEM-driven DT systems enable virtual testing of different designs without relying on expensive real physical prototypes. Engineers can optimize designs for weight reduction, structural integrity, and performance by running multiple FEM simulations. Furthermore, FEM allows DT to simulate extreme operating conditions, such as high loads, impacts, or thermal shocks, ensuring safety validation and compliance with standards and regulations.

**Figure 2:**
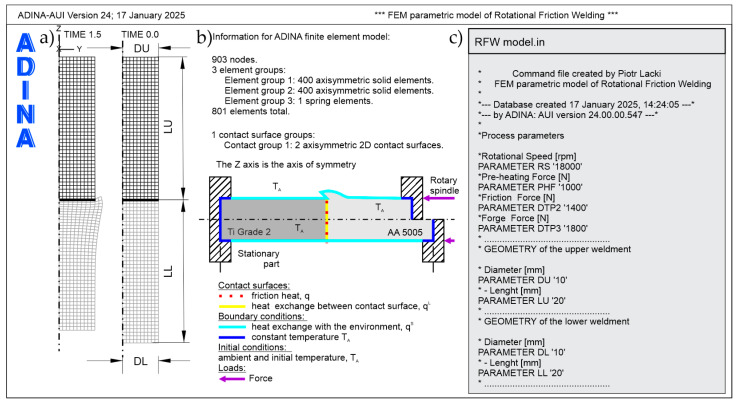
Parametric FEM model; (**a**) finite element mesh; (**b**) boundary and initial conditions; (**c**) part of the parametric model file.

FEM results can be used to train Machine Learning (ML) algorithms within Digital Twin (DT) systems, enhancing predictive accuracy. Figure 2 presents the developed parametric numerical FEM model, which is used to train the artificial neural network (ANN). The model accounts for a wide range of variable parameters, particularly for the Ti Grade 2/AA 5005 friction pair. It is proposed to adopt a temperature-dependent friction coefficient, as described in detail in [1]. The friction coefficient is a material property that determines the efficiency of converting mechanical work into heat. A higher friction coefficient leads to more effective heat generation, thereby increasing the peak temperature. The friction coefficient may vary with temperature, material condition, and surface cleanliness. Materials with higher friction coefficients typically achieve higher peak temperatures during RFW.

Materials with low thermal conductivity (Ti Grade 2) are slower to remove heat from the faying surface, which leads to higher maximum temperatures in the RFW welding temperature range of the Ti Grade 2/AA 5005 joint (380–600 °C). In contrast, materials with high thermal conductivity (AA 5005) dissipate heat more rapidly, reducing peak temperatures. Materials with higher heat capacity require more heat input to achieve the same temperature rise, which can lower the maximum temperature for a given set of parameters. Materials with lower flow stress at elevated temperatures deform more easily, potentially reducing frictional heating as material deformation dominates over sliding. The geometry and surface roughness of the components influence the initial contact conditions, which, in turn, affect heat generation. Rougher surfaces may increase initial friction, resulting in higher temperatures, while smoother surfaces may reduce heat generation at the interface. Heat dissipation through the surrounding material and environment can limit the maximum temperature. Materials with higher thermal conductivity or external cooling systems dissipate heat more efficiently, reducing peak temperatures. By combining FEM with AI, DT can predict complex behaviors without requiring full-scale FEM simulations each time.

### 2.2. DT Based on ANN

DT uses ANN to learn relationships between inputs (friction force and rotational speed) and outputs (temperature) based on historical and real-time data. This enables rapid simulations without the need to solve complex physical equations. ANN can work alongside traditional models (e.g., FEM) to enable efficient simulations and address gaps where physical models are incomplete. Hybrid models combine data-driven learning with physics-based constraints to create more accurate and efficient simulations. In the work [14], an ANN-based incremental learning system was developed to predict crack growth and fatigue in the RFW joints of AA5083 and AA7075 aluminum alloys. The results demonstrate that this method can effectively accommodate input temperatures and the S-N curve, providing reliable predictions of expected fatigue. This approach can reduce costs and time required for crack propagation tests, thereby enhancing production processes and reducing overall costs. Furthermore, it shows promise for extrapolating monotonic functions, paving the way for solving various predictive problems. Additionally, verification using AISI 2205 and AISI 1020 steel showed that neural networks could obtain S-N curve values for other metals with an error rate of less than 8%.

**Figure 3:**
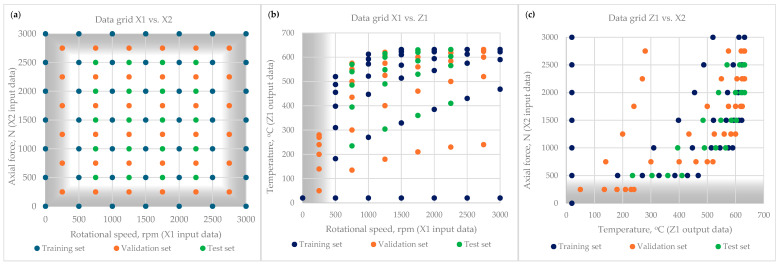
Scatter plot of the data grid for three subsets in different configurations; (**a**) rotational speed vs. axial force; (**b**) rotational speed vs. temperature; (**c**) temperature vs. axial force.

The detailed scope of the numerical data used to analyze the ANN is presented in Table A1 of Appendix A. Figure 3 illustrates the data grid, divided into three sets: (a) Training Set, (b) Validation Set, and (c) Test Set. Before training the neural network, a representative dataset was generated that accurately reflects the problem domain. The dataset from the FEM calculation contained 105 cases, divided into three subsets: training set (47%), validation set (36%), and test set (20%). This dataset size was optimal for network performance. The data also included scenarios that, while not practically relevant, were still useful for the analysis. All features contribute equally to the task. Care was taken to ensure that each data sample in each set had the same probability of occurrence.

Each subset plays a crucial role in ensuring effective learning and generalization to unseen data. The data obtained from the FEM model are consistent, and no augmentation, dropout, or weight decay techniques were applied. Only the normalization technique was applied to the FEM simulation data before using them in neural network training. The RFW process parameters not used in the analyzed case but theoretically possible to use, which were calculated by FEM equations, are marked in gray in Figure 3.

Designing a neural network topology involves choosing the number of hidden layers, the number of neurons per layer, and the activation functions based on the problem’s complexity. In general, the selection of the topology depends on the nature of the data and the target function. In the analyzed case, note that the network comprises two input neurons and one output neuron, with nonlinear dependencies between the input and output data. Therefore, the problem cannot be solved using a simple linear model; it requires nonlinear transformations. The input data interact in a complex manner that requires at least one hidden layer. There is no fixed formula for determining the number of layers or the number of neurons per hidden layer. The practical approach in this work assumes that a single hidden layer (a 2-H-1 configuration) is often sufficient for most nonlinear problems, although two hidden layers (a 2-H1-H2-1 configuration) may be required for more complex relations. To determine the starting number of neurons, an empirical formula was employed:

(1) $$H=\frac{N_{in}+N_{out}}{2}+k,$$

where *H* is the number of hidden neurons, *N_in_* is the number of inputs (2), *N_out_* is the number of outputs (1), and k is a small integer (from 2 to 10). Example: *H* = (2 + 1)/2 + 7 = 8, so we start with 8 neurons in the hidden layer.

**Figure 4:**
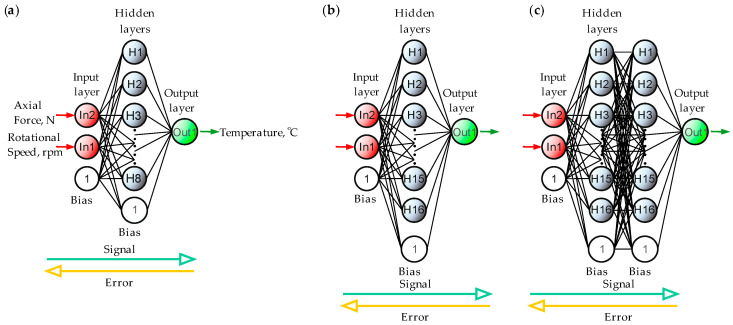
Applied ANN topologies; (**a**) topology: 2-8-1; (**b**) topology 2-16-1; (**c**) topology 2-16-16-1.

The initial 2-8-2 topology was expanded by increasing the hidden layer to 16 neurons (H = 16), and then a second hidden layer with 16 neurons was added. Figure 4 presents the ANN topologies used in the work.

Activation functions introduce nonlinearity into neural networks, enabling them to model complex relationships in data. In this work, three types of activation functions are used: Linear, Hyperbolic Tangent (Tanh), and Sigmoid (or Logistic). The linear activation function is applied at the output of the input layer to evenly distribute the signal to all neurons in the first hidden layer. It is the simplest and most computationally efficient, as its output is directly proportional to its input:

(2) f(x)=x,

Range: (−∞,+∞); Derivative: *f*′(*x*) = 1 (constant)

The Tanh activation function is a scaled version of the Sigmoid function that outputs values in the range (−1, 1). It is characterized by a zero-centered output, which makes training more stable than using the Sigmoid function. The Tanh function provides better nonlinearity than a linear activation and works well for hidden layers in neural networks, especially for problems where the data are centered around zero. However, due to its shape, it is computationally more expensive than the linear activation function:

(3) $$f\left(x\right)=\frac{2}{1+e^{-2x}}-1,$$

Range: (−1, 1); Derivative: *f*′(*x*) = 1 − *tanh*^2^(*x*)

The Sigmoid function is a nonlinear function that squashes input values to the range (0, 1). It is well-suited for binary classification problems because its output can be interpreted as a probability. While it is differentiable and smooth, for very large or very small input values, the gradients approach zero, which slows down training:

(4) $$f\left(x\right)=\frac{1}{1+e^{-x}},$$

Range: (0, 1); Derivative: *f*′(*x*) = *f*(*x*)(1 − *f*(*x*))

The learning rate α is a crucial hyperparameter in neural networks that controls the step size at which the model updates its weights during training. It determines how much the model learns in each iteration and significantly affects convergence speed, performance, and stability. In this work, depending on the network model, the learning rate was set in the range from α = 0.1 to 0.0001. An optimal learning coefficient was selected to balance speed and stability while enabling efficient convergence without overshooting. If α is too high, weight updates become too large and the model may never converge; conversely, if α is too low, training becomes very slow and may get stuck in local minima. In this work, one full pass through the dataset (i.e., one epoch) ranged from 100 to 10,000 iterations, depending on the network model, while the batch size was set to 1.

Statistical metrics describing individual ANN cases and the hyperparameters adjusted to obtain the optimal ANN are presented in Table 1.

In the applied neural network model, Mean Squared Error (MSE) was used as the loss function. MSE measures the average squared difference between the actual and predicted values and helps train the network by minimizing errors via gradient descent. A lower MSE indicates more accurate predictions, whereas a higher MSE suggests larger errors. Since MSE squares the errors, it penalizes large deviations more than small ones, making it well-suited for minimizing errors during ANN training. In addition, R-squared (R^2^) was used to evaluate the model’s performance after training. R^2^ is a statistical metric that assesses how well the model’s predictions match the actual values by measuring the proportion of variance in the dependent variable explained by the independent variables. Unlike MSE, which measures absolute error, R^2^ indicates how well the model generalizes, facilitating comparisons between different models. Both MSE and R^2^ were analyzed together to ensure a well-performing regression model.

The last column of Table 1 provides the training time for individual ANNs. The training time of an ANN depends on multiple factors, including the number of epochs, batch size, network complexity, dataset size, and available computational power. The number of epochs used (ranging from 100 to 10,000) were sufficient to ensure learning while avoiding overfitting. Early stopping was employed to determine the optimal training point. Batch size affects training stability and speed; smaller batch sizes lead to more frequent updates, whereas larger batch sizes require more memory. A fixed batch size of 1 was used. Network complexity, defined by the number of layers and neurons, increases training time but enhances model capacity, requiring techniques such as dropout and batch normalization for efficiency. Large datasets and high-dimensional data require more processing but improve generalization, often benefiting from dimensionality reduction methods. Finally, computational resources such as GPUs and TPUs significantly accelerate training compared to CPUs.

### 2.3. GA-Based Optimization

GA are powerful optimization tools inspired by the principles of natural selection and evolution. They are well-suited for solving complex, nonlinear, and multi-objective optimization problems, such as those encountered in the RFW process. By integrating GA with the Digital Twin (DT) of the RFW process, it is possible to efficiently explore a large parameter space and identify optimal welding conditions to improve joint quality, minimize defects and maximize process efficiency. GA solves multi-criteria problems balancing goals such as cost, performance, energy efficiency, and durability. DTs use Gas to find trade-offs between conflicting objectives. Gas assist DTs in calibrating system models by automatically tuning parameters to match real-world behavior, resulting in more accurate and realistic simulations. They allow DTs to continuously evolve and adapt their models over time as new data are collected, thereby improving decision-making and system resilience. Gas can find global optima in complex, nonlinear, and multi-modal problem spaces. They handle both discrete and continuous variables, evaluating multiple solutions simultaneously to speed up optimization. Unlike gradient-based methods, Gas do not require differentiable models, making them ideal for black-box optimization. By integrating Gas, DTs become highly adaptive and have the capability to discover innovative solutions that drive efficiency, performance, and resilience across industries.

In [15], a GA was used to optimize the parameters of the RFW process for dissimilar light alloys (AZ31B/AA7075). The optimized values were compared with experimental results, and the GA method increased the tensile strength of the joint from 88 to 180 MPa. A similar approach to optimize the parameters of the RFW process was presented in [16]. Parameters such as spindle speed, friction pressure, upset pressure, and burn-off length were optimized to achieve maximum Ultimate Tensile Strength (UTS) and hardness for the aluminum metal-matrix composite AA7075-10 vol% SiCP-T6 using GA with a Combined Objective Function (COF). The main advantage of using GA is that it converges to optimized values for various weighting factors in a minimal number of iterations, making it suitable for solving problems with a large search space, such as friction welding. The strong correlation between the optimized and validated results (in terms of tensile strength and hardness) demonstrates the method’s accuracy in achieving the objective.

The first step in applying genetic algorithms to RFW optimization is to formulate a Fitness Function (*F*) that quantifies the quality of the weld. The proposed optimization function includes parameters such as: maximizing joint strength (e.g., tensile strength, fatigue resistance); minimizing defects (e.g., voids, cracks, excessive flow); optimizing temperature distribution to prevent unwanted intermetallic formation in dissimilar welding; minimizing residual stresses and deformations; and reducing cost or energy consumption.

A weighted sum function was used to optimize the RFW process, considering several qualitative or quantitative criteria into a single *F* with appropriate weighting factors. An *F* with penalties was added to account for additional constraints (e.g., maximum temperature, minimum strength, limited intermetallic layer thickness). In this approach, *F* is extended with components that “penalize” solutions violating these constraints. The *F* proposed for the optimization of the RFW process can be mathematically expressed as:

(5) $$F=\sum_{i=1}^{n}{\omega_{i}\cdot f}_{i}(x)+\sum_{i=1}^{m}\lambda_{i}\cdot max\left\{0,\;g_{i}(x)\right\}$$

where:

*ω_i_*—weighting coefficients defining the relative importance of individual criteria,

*f_i_*(*x*)—individual objective functions (e.g., maximizing strength, minimizing defects, minimizing temperature differences, etc.),

*λ_i_*—penalty coefficients that determine how much violating a constraint affects the value of the objective function,

*g_i_*(*x*)—constraint functions (may take positive values when the constraint is violated).

In the adopted F, each criterion is normalized to a common scale to ensure that their impacts are properly compared. The F value is calculated as the sum of the products of individual criteria and their weights. Solutions that satisfy all constraints receive no penalties, so the *F* value equals *f*(*x*). If any constraint is violated, a penalty is added to the objective function, which deteriorates the evaluation of the solution. Optimization involves finding a set of parameters that minimizes or maximizes the function *F*—depending on the definition—while meeting technical and qualitative criteria. The selection of appropriate weights requires preliminary research or experimentation. In some cases, the function may not fully reflect the trade-offs between criteria, particularly when dealing with strongly divergent scales or conflicting goals.

GA requires encoding the RFW parameters into a chromosome-like structure. The following coding scheme was adopted:

*Chromosome* = [*N*, *F_A_*, *t_f_*] (6)

where: *N* = Rotational speed, rpm; *F_A_* = Axial force, *N*; *t_f_* = Friction time, s.

A population (PS) of RFW parameter sets is randomly generated, with each individual representing a different combination of parameters. Each individual is evaluated using the objective function, and the Fitness Score (FS) indicates how well its parameters fit the *F*. Based on the FS, the best-performing individuals are selected using a Selection Method (SM) technique. In this paper, a tournament selection strategy is employed; the Tournament Size (TS) specifies the number of individuals randomly selected to compete for selection, with larger sizes increasing selection pressure. To explore new solutions, the selected individuals undergo crossover, during which parts of their parameter sets are swapped to produce offspring with mixed traits. The Crossover Rate (CR) indicates the probability that two parental chromosomes will undergo crossover to produce offspring; a higher CR promotes the exploration of new solutions. To introduce diversity and avoid premature convergence, a Mutation Rate (MR) is applied to randomly alter some parameters. The MR represents the probability that individual genes (parameters) will be changed during mutation, which is crucial for maintaining diversity and avoiding local minima, thereby ensuring that the algorithm explores a wider search space. The Number of Generations (NG) denotes the total number of iterations the GA will perform to develop solutions. The process repeats over multiple generations, refining the parameters to reach an optimal solution. Convergence occurs when the fitness function stabilizes, meaning that further iterations produce no significant improvements. The Elitism Rate (ER) indicates the proportion of the best individuals preserved into the next generation without modification, helping to retain high-quality solutions across generations. The Termination Criteria (TC) specify the conditions under which the GA stops running (e.g., reaching the maximum number of generations or a convergence threshold), thereby preventing unnecessary computation once an optimal solution is reached. Finally, the Encoding Scheme (EC) employs a real-valued representation of decision variables, which is consistent with the nature of the problem parameters.

## 3. Results

**Figure 5:**
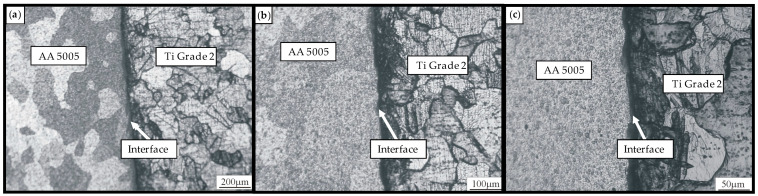
Welded interface at different magnifications; (**a**) low magnification; (**b**) medium magnification, (**c**) high magnification.

The microstructure of dissimilar joints between Ti Grade 2 and AA 5005 aluminum alloy, obtained through RFW, reveals distinct regions with characteristic features depending on the magnification level. Observing the welded interface at different magnifications, as shown in Figure 5, facilitates the analysis of metallurgical changes, intermetallic phase formation, and material flow behavior. Microstructure images were captured for a sample joint produced at a rotational speed of n = 1440 rpm and an axial force of F_A_ = 1000 N. However, the following discussion applies to the entire scope of the study.

At low magnification Figure 5a, the microstructure provides a macroscopic view of the welded joint, highlighting the overall morphology of the interface between Ti Grade 2 and AA 5005. Flash formation due to excessive material flow may also be evident, particularly on the aluminum side, given its lower strength and higher deformability. The heat-affected zone (HAZ) appears as a transition region near the interface, with the aluminum side typically showing a larger HAZ due to its higher thermal conductivity and lower melting point. In contrast, titanium exhibits minimal deformation due to its higher strength and lower thermal conductivity, resulting in a narrower HAZ.

At medium magnification Figure 5b, the microstructure reveals more detailed features of the interface and adjacent regions. The titanium side near the interface retains a refined, equiaxed grain structure, while its small HAZ shows slight grain elongation or refinement depending on the distance from the weld interface. In the aluminum alloy, significant grain refinement occurs due to dynamic recrystallization, leading to smaller and more uniform grains in the HAZ compared to the base material. Evidence of plastic flow is visible as elongated grains near the interface, indicating material mixing and deformation during welding. A thin intermetallic compound (IMC) layer may also be present at the interface, formed due to high temperatures and chemical interaction between titanium and aluminum. Ensuring controlled plastic deformation and uniform material flow, as well as managing heat generation along the radial direction, helps minimize harmful compounds and improve the uniformity of the joint’s mechanical properties. The formation of IMCs at the interface is primarily influenced by welding parameters such as friction load, burn length, and upsetting load [1].

At high magnification Figure 5c, finer details of the interface and intermetallic compounds become visible. IMC thickness increases with higher temperatures or prolonged friction time, potentially leading to joint embrittlement if excessive. Any microvoids, cracks, or discontinuities at the interface can be observed, often resulting from improper welding parameters or uneven material flow. The presence of oxide layers or impurities (such as titanium oxide and aluminum oxide) may also be detected, particularly if pre-welding surface treatment was inadequate. Small regions of aluminum may become embedded in the titanium surface due to high-pressure contact and deformation, while titanium fragments may appear in the aluminum matrix, forming a mixed zone near the interface. Grain refinement on both sides enhances mechanical properties, particularly tensile strength and fatigue resistance. However, defects such as micro voids, cracks, or excessive IMC formation can compromise joint performance. This multiscale analysis underscores the importance of carefully controlling RFW parameters to optimize microstructure and ensure a robust joint between Ti Grade 2 and AA 5005.

### 3.1. FEM Results

**Figure 6:**
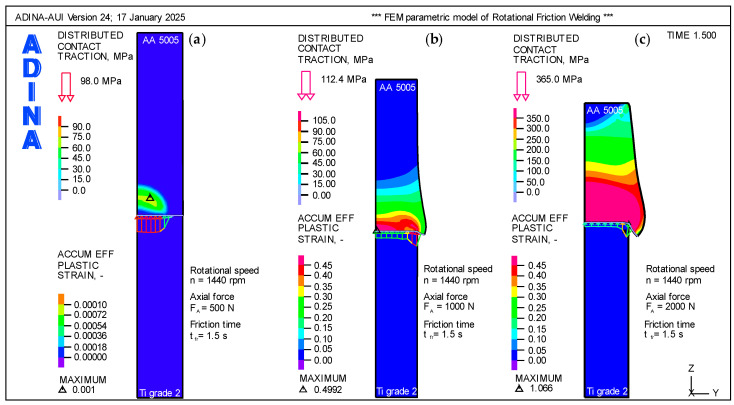
Plastic strains as a function of axial forces, N; (**a**) F_A_ = 500 N; (**b**) F_A_ = 1000 N; (**c**) F_A_ = 2000 N.

The distribution of plastic strain in the weldment volume during RFW process of a Ti Grade 2 rod and an AA 5005 aluminum alloy rod depends on the axial force FA applied during the process. Higher axial forces lead to greater plastic deformation due to increased contact pressure, material flow, and heat generation. Figure 6 presents a quantitative analysis of plastic strain distribution for different axial force values (F_A_ = 500 N, 1000 N, and 2000 N).

At a low axial force Figure 6a; F_A_ = 500 N, frictional heat generation is relatively low, resulting in limited thermal softening and material flow. The maximum plastic strain (ε_max_ = 0.001) is localized near the interface on the aluminum alloy (AA 5005) side, due to its lower strength and higher plasticity compared with titanium, which exhibits minimal deformation due to its high yield strength and stiffness. The HAZ is small, with minimal material flow or deformation outside the immediate interface region. The aluminum side exhibits small plastic strains near the interface, whereas the titanium side remains largely unaffected. Weak material flow on the aluminum side results in less pronounced flash formation, which may lead to an incomplete joint.

At an optimal axial force Figure 6b; F_A_ = 1000 N the plastic strain at the interface becomes more pronounced (ε_max_ = 0.4992), and the aluminum alloy undergoes significant plastic flow due to increased pressure and heat generation. Titanium begins to exhibit slight plastic deformation near the interface, primarily due to higher temperatures and increased contact pressure. Material flow significantly improves on the aluminum side, leading to noticeable flash formation. However, titanium still exhibits limited material flow due to its higher stiffness and lower thermal conductivity.

At a high axial force Figure 6c; F_A_ = 2000 N plastic strain increases substantially (ε_max_ = 1.066) on the aluminum side. The aluminum alloy undergoes severe plastic flow, with material being extruded as flash. However, the titanium side does not undergo noticeable plastic strain near the interface despite the increased heat and pressure. Excessive axial force may lead to overheating and the formation of thick intermetallic layers (e.g., TiAl_3_) at the interface, which can reduce joint strength due to brittleness.

**Figure 7:**
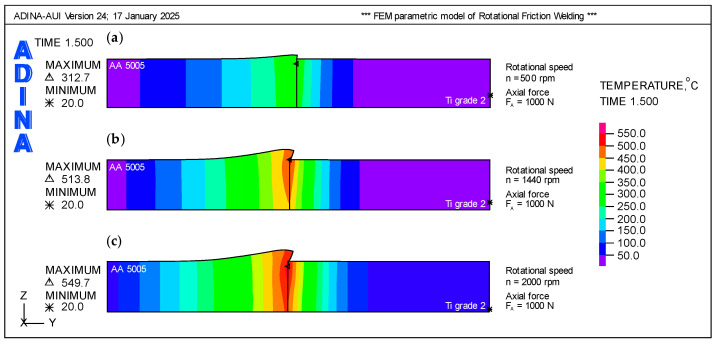
Temperature, °C as a function of rotational speed, rpm; (**a**) n = 500 rpm; (**b**) n = 1440 rpm; (**c**) n = 2000 rpm.

The temperature distribution in the weldment volume during the RFW process of a Ti Grade 2 rod and an AA 5005 aluminum alloy rod depends on frictional heat generation at the faying surfaces, the thermal conductivity of the materials, and heat dissipation into the surrounding volume. Figure 7 illustrates how the temperature distribution varies at different rotational speeds.

At low rotational speeds Figure 7a; n = 500 rpm, the relative motion at the interface generates limited frictional heat. The peak temperature (T_max_ = 312 °C) at the interface remains relatively low—insufficient to fully soften or plasticize the materials. Heat is concentrated at the immediate interface, with a steep temperature gradient extending into both materials. The aluminum alloy (AA 5005) side experiences a greater temperature rise than the titanium (Ti Grade 2) side, due to aluminum’s lower thermal conductivity. The HAZ is narrow, and insufficient thermal energy may lead to weak bonding.

At optimal rotational speeds Figure 7b; n = 1440 rpm, sufficient frictional heat is generated, raising the interface peak temperature to T_max_ = 513 °C. The aluminum alloy softens and may approach its melting range, while titanium remains solid but thermally softened. The interface reaches higher temperatures, promoting material flow and enhanced mixing of the dissimilar metals. Due to its lower thermal conductivity, the aluminum alloy side experiences a greater temperature gradient and deeper heat penetration. In contrast, the titanium side undergoes a moderate temperature increase, but its thermal gradient remains steeper compared to aluminum. The heat-affected zone (HAZ) is larger than at lower speeds, enhancing joint quality.

At high rotational speeds Figure 7c; n = 2000 rpm) frictional heat generation becomes intense, raising the interface peak temperature to T_max_ = 549 °C. The aluminum alloy may reach or exceed its melting point, while titanium remains in a solid state due to its higher melting temperature. However, excessive heat at high speeds may lead to undesirable effects, such as the formation of brittle intermetallic compounds (e.g., TiAl_3_) or defects resulting from material over-softening on the aluminum side.

### 3.2. ANN Results

The result of an ANN is a set of predicted outputs generated from input data after training. The accuracy and reliability of these predictions depend on the quality of the training data, network architecture, and optimization process. When trained correctly, the ANN approximates complex relationships within the data, producing outputs that closely match expected values. The ANN’s performance is typically evaluated using statistical metrics such as MSE and R^2^, which quantify the difference between predicted and actual values.

As part of the analysis, eight cases (ID 1–8) were examined, as shown in Table 1. The individual networks differ primarily in topology and the number of neurons, while activation functions also play a crucial role. A linear activation function was applied at the output of each input layer. The ID 1–8 networks exhibit a progressive increase in complexity. Beginning with ID 1, each subsequent network demonstrates higher accuracy. The networks evolve from a simple 2-8-1 structure to more complex 2-16-1 and 2-16-16-1 architectures, highlighting the trade-off between complexity, performance, and training time.

**Figure 8:**
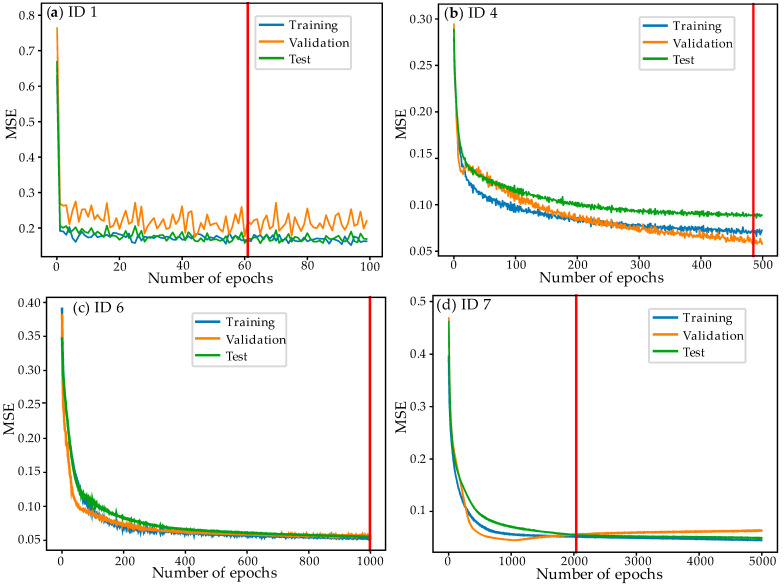
Variability of MSE for different ANN models as a function of the number of epochs; (**a**) ID 1; (**b**) ID 4; (**c**) ID 6; (**d**) ID 7.

Figure 8 presents three plots of MSE versus epochs for representative ANN models. Model (a), ID 1 (2-8-1, Linear-Linear-Linear, LR = 0.1, 100 epochs), shows rapid error reduction in the initial epochs but exhibits significant fluctuations, particularly in validation MSE. While training and test MSE remain relatively stable, the validation MSE oscillates, indicating poor generalization. The model stops early (epoch 61), suggesting early plateauing or overfitting. The linear activation functions restrict complexity, making the model less effective at capturing intricate patterns. Although the model learns quickly, it lacks expressive power, resulting in greater error variance.

The simplest model utilizes linear activation functions throughout and a high learning rate of 0.1, achieving moderate performance with MSE = 0.157 and R^2^ = 0.6 in under four seconds. As the architecture expands to a 2-16-1 configuration, and activation functions are adjusted—initially using a Sigmoid output, then incorporating a Tanh hidden layer followed by a Sigmoid output—the network achieves higher accuracy with reduced MSE and increased R^2^ values, albeit with longer training times. The transition to a deeper 2-16-16-1 network, incorporating two hidden layers with Tanh activation functions and a Sigmoid output, further enhances predictive capability, achieving lower error metrics and higher R^2^, particularly when the learning rate is lowered to 0.001 or 0.0001 and epochs are increased. However, this improvement comes at the cost of significantly longer training times. Overall, the transition from a simple to a more complex network illustrates how increasing neurons and layers, fine-tuning activation functions, and adjusting learning rates and epochs enhance the approximation of FEM data, while also highlighting the inherent trade-offs between accuracy and computational efficiency.

In case (b), ID 4 (2-16-1, Linear-Tanh-Sigmoid, LR = 0.01, 500 epochs), the MSE decreases smoothly during the initial epochs, indicating stable learning. Using the Tanh activation function in the hidden layer enhances nonlinearity, enabling better representation of complex relationships. Training proceeds until epoch 485, demonstrating continuous improvement with additional epochs. The validation and test MSE remain close to the training MSE, suggesting a good balance between learning and generalization. This network is better optimized than the previous one, showing enhanced nonlinearity, lower MSE, and stable training.

In the best case (c), ID 7 (2-16-16-1, Linear-Tanh-Tanh-Sigmoid, LR = 0.001, 5000 epochs), the MSE gradually decreases due to the small learning rate (0.001). Training continues until epoch 2038, after which the validation/test MSE starts to increase slightly, indicating potential overfitting. The deeper architecture with two hidden layers utilizing Tanh activation functions enhances feature extraction, resulting in the lowest MSE. The longer training time is a result of the lower learning rate and deeper network structure. While the deep network achieves high accuracy, it requires significantly more epochs, and overtraining may lead to overfitting.

**Figure 9:**
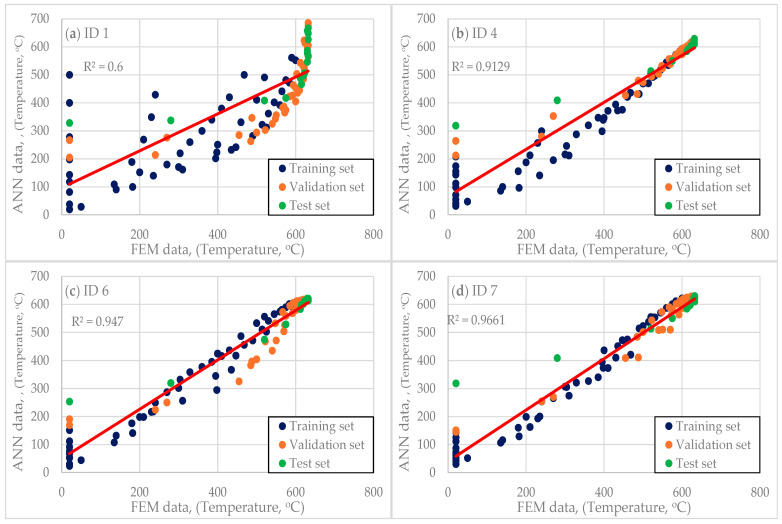
Scatter plots of FEM vs. ANN data for the network architectures; (**a**) ID 1; (**b**) ID 4; (**c**) ID 6; (**d**) ID 7.

The scatter plots In Figure 9 compare FEM data with ANN predictions for different network architectures. The ANN model’s quality is determined by how closely its predicted values align with FEM data along the red regression line and its corresponding R^2^ value. For the shallow ID 1 network, R^2^ = 0.6. The points deviate significantly from the regression line, particularly for low and midrange temperatures. The widely scattered pattern of the data indicates higher prediction errors. The linear activation functions limit the model’s ability to capture complex relationships, reducing performance. This model demonstrates moderate accuracy but struggles with nonlinearity, leading to noticeable deviations.

The network with architecture ID 4 has an R^2^ value of 0.9129. Its alignment with the regression line is significantly improved, exhibiting lower scatter and variance. The Tanh activation function enhances the model’s ability to capture nonlinear patterns, improving accuracy compared to ID 1. However, some deviations persist for midrange temperature values, suggesting slight underfitting. This model achieves higher accuracy, demonstrating a more effective mapping between FEM and ANN predictions.

The deep network ID 7 achieves the highest R^2^ value of 0.9661 among the analyzed architectures. The data points are closer to the regression line than in ID 4, indicating high accuracy. Minimal scatter indicates strong generalization across different datasets (training, validation, and test). The increased network depth and lower learning rate enable more precise predictions. This model provides the best fit by effectively capturing FEM data patterns and generating accurate predictions.

**Figure 10:**
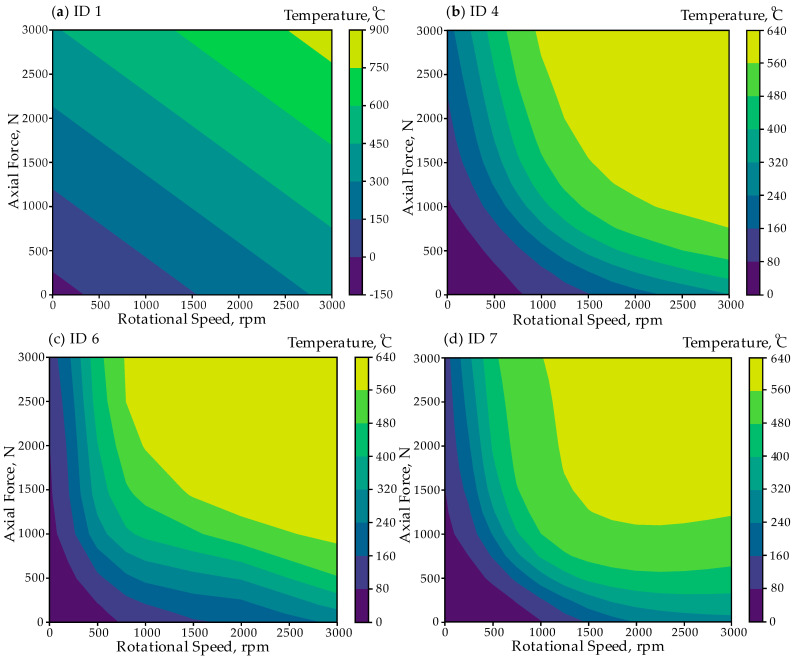
Temperature distribution prediction, °C by ANN; (**a**) ID 1; (**b**) ID 4; (**c**) ID 6; (**d**) ID 7.

The analysis of temperature distribution predictions for the examined network architectures is best illustrated through contour plots. Figure 10 presents contour plots depicting the temperature distribution as a function of axial force, N, and rotational speed, rpm for three ANN models. The accuracy of these predictions depends on network depth, activation functions, and training quality.

For the shallow ID 1 network, the temperature distribution appears oversimplified, exhibiting linear gradient patterns. The temperature isolines appear as straight lines, indicating poor generalization. Unrealistic temperature values appear in the color bar, ranging from −150 °C to 900 °C, significantly deviating from expected physical trends. This model fails to capture nonlinear relationships, resulting in unrealistic and unreliable predictions.

For the ID 4 network, the temperature distribution appears more structured, with smooth and continuous transitions. The high-temperature region (yellow) is concentrated at higher rotational speeds and moderate axial forces, aligning with expected physical trends. The contour lines indicate a better representation of nonlinearities, resulting in more realistic predictions. This model performs significantly better than ID 1, handling complex relationships more effectively, although some deviations persist.

The ID 7 network exhibits the smoothest temperature distribution. The high-temperature region is well-defined and physically consistent. The model captures clearer boundary transitions, enhancing prediction accuracy. Its temperature distribution aligns most closely with expected FEM results, suggesting better generalization and learning capability. ID 7 is the most accurate and physically consistent model, effectively predicting realistic temperature variations.

### 3.3. GA Result

Each fitness function variant (A–H) in Table 2 represents a unique method for integrating RFW parameters (n, F_A_, T) and temperature constraints into a single metric, minimized by the genetic algorithm. Temperature constraints, ensuring the temperature remains within specified limits, are crucial for preventing poor bonding (too low) or excessive intermetallic formation (too high). The fitness functions combine n, F_A_, and T in various ways to capture different aspects of the welding process—such as energy input, mechanical stability, or final joint quality.

All fitness functions are subject to minimization. Typically, a lower function value indicates an optimal set of parameters (e.g., minimal temperature deviation, minimal defect formation, or minimal energy consumption). The differences lie in how temperature is penalized or rewarded, how rotational speed and axial force are scaled or combined, and how constraints are enforced.

These differences, along with varying GA hyperparameters (population size, selection method, and mutation/crossover rates), facilitate a systematic exploration to determine the optimal RFW settings. Variants with complex or multi-objective fitness definitions required larger populations and more generations for reliable convergence.

Ultimately, selecting a fitness function depends on optimization goals—whether focusing on defect reduction, mechanical performance, energy efficiency, or a balanced approach.

## 4. Discussion

ANNs play a transformative role in the creation and operation of DT by enabling them to learn, adapt, and make accurate predictions based on vast datasets. ANNs complement traditional physics-based models, such as the FEM, by offering data-driven insights, improving efficiency, and enhancing real-time decision-making. They are particularly effective in modeling complex, nonlinear systems where physical modeling is challenging or computationally expensive. ANNs excel at recognizing patterns in large datasets, allowing DT to predict equipment failures or performance degradation. By analyzing sensor data, ANNs can forecast maintenance needs, reducing downtime and costs. Additionally, they enable DT to make real-time adjustments for optimized system performance by continuously learning from operational data and recommending changes to improve efficiency, energy consumption, or output.

To validate a neural network trained on FEM data, various evaluation techniques should be employed, including statistical metrics (MSE, R^2^, and error analysis), graphical comparisons (scatter plots and residuals), and sensitivity tests. An optimized ANN should exhibit low errors, high R^2^ values, and close alignment with FEM results. If discrepancies persist, refining the training process, increasing data diversity, or adjusting the network architecture may be necessary.

**Figure 11:**
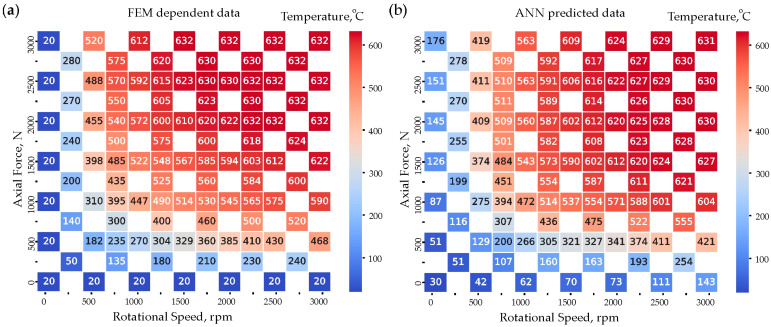
Comparison of annotated heatmaps for; (**a**) FEM-dependent data; (**b**) ANN-predicted data.

Heatmaps in Figure 11 illustrate the temperature distribution as a function of axial force, N and rotational speed, rpm. The left heatmap represents FEM-dependent data, while the right heatmap displays ANN-predicted data. The temperature distribution in FEM-dependent data follows an orderly and physically consistent trend, with higher temperatures occurring at higher rotational speeds and axial forces. The values are well-organized, increasing smoothly from bottom-left (low force/speed) to top-right (high force/speed). The highest recorded temperature, T_max_ = 632 °C, is in the upper-right region, indicating maximum thermal impact. This dataset serves as the ground truth reference for evaluating ANN predictions.

**Figure 12:**
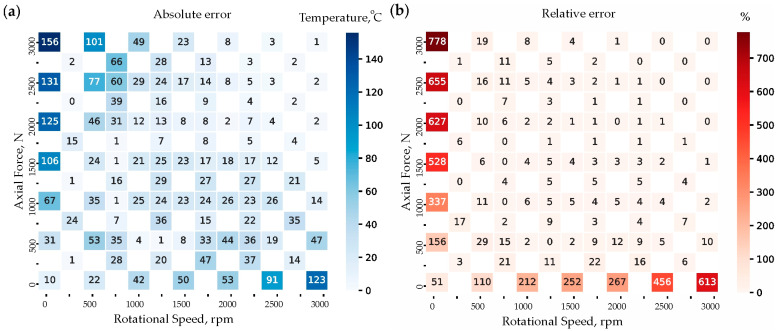
Statistical and graphical evaluation of the similarity between FEM and ANN predictions; (**a**) Absolute error, °C; (**b**) Relative error, %.

The ANN model captures the general trend observed in the FEM data but exhibits some deviations. In low axial force and low rotational speed regions, the ANN underestimates temperatures slightly. Conversely, in higher rotational speed and force regions, ANN predictions closely align with FEM values, demonstrating a good approximation of the physical behavior. Minor inconsistencies are present, such as slight overestimation or underestimation in midrange temperatures, highlighting areas for potential improvement. Further optimization of the network architecture, training data, or hyperparameters could enhance the ANN’s performance and minimize deviations. While ANNs provide a strong but imperfect approximation of FEM results, they remain a valuable yet improvable tool for predicting temperature distributions. Figure 12 presents a comparative error analysis between FEM data and ANN predictions, using absolute error (°C) and relative error (%). These heatmaps provide insights into how well the ANN model approximates FEM-generated temperature data across various axial force (N) and rotational speed (rpm) combinations. The absolute error represents the direct numerical difference between FEM and ANN temperature predictions. Most values remain low, which indicates the ANN’s effectiveness in approximating FEM data. However, higher errors (above 100 °C) are observed at extreme values of axial force (e.g., 3000 N) and rotational speed. The majority of midrange values (1000–2500 rpm, 500–2000 N) have errors below 50 °C, suggesting good ANN generalization in these regions. This analysis indicates that ANN errors tend to increase at high forces and speeds, potentially due to nonlinearities in thermal behavior.

The relative error (%) highlights the proportional deviation between the ANN predictions and the FEM results. Higher relative errors occur in regions with low temperatures (20–200 °C), especially at axial forces (0–500 N) and low rotational speeds (0–500 rpm), where the temperatures are small, making any deviation appear large in percentage terms. Some extreme values (e.g., 613% at n = 3000 rpm, FA = 0 N and 778% at n = 0 rpm, FA = 3000 N) arise due to the ANN incorrectly predictsthe temperatures (143 °C and 176 °C, respectively) in these regions, making the small absolute deviations appear disproportionately large in percentage terms.

Extreme values of both absolute and relative error occur at high force and low speed, as well as low force and high speed. The ANN performs well in mid-range values (n = 1000–2500 rpm, F_A_ = 500–2000 N) but struggles at extreme conditions. The primary source of error likely stems from the neural network’s limitations in capturing nonlinear heat distribution. Future refinements, such as incorporating more training data in low-temperature regions, could further reduce prediction errors.

**Figure 13:**
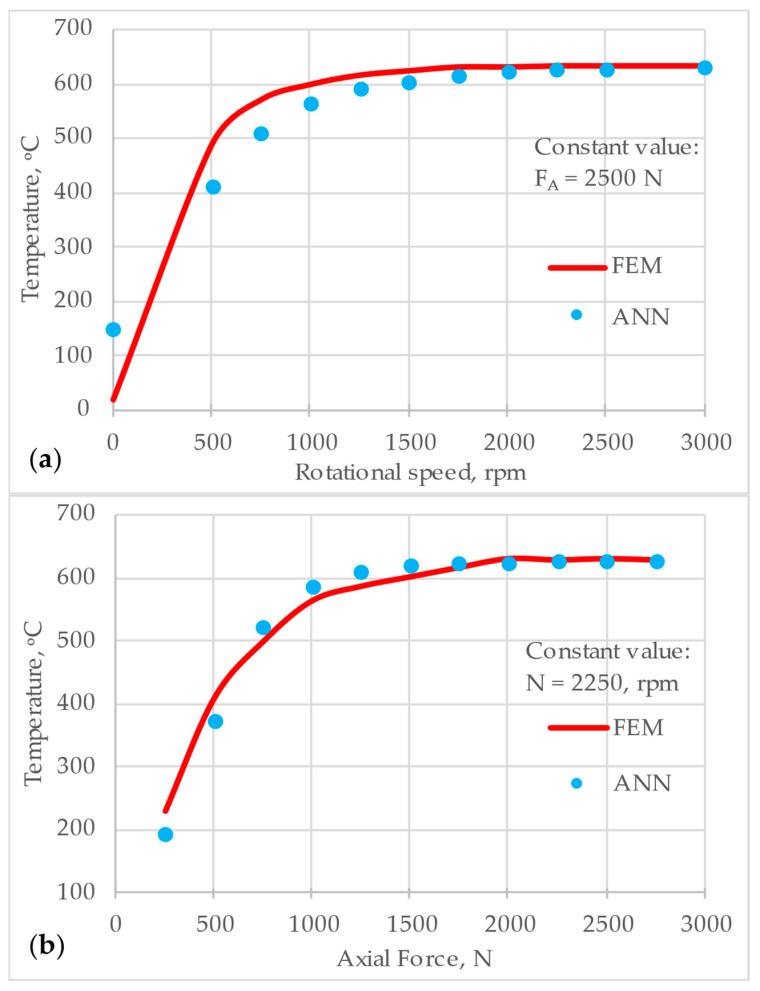
Temperature prediction for ANN ID 7 network architecture; (**a**) Rotational Speed, rpm vs. Temperature, °C at constant axial force F_A_ = 2500 N; (**b**) Axial Force, N vs. Temperature °C at constant rotational speed n = 2250 rpm.

Figure 13 compares temperature predictions from the ANN ID 7 architecture (blue dots) with reference FEM data (red curve) in two scenarios. Case (a) presents varying rotational speed at a constant axial force (F_A_ = 2500 N). As the rotational speed increases, both FEM data and ANN predictions exhibit a rapid rise in temperature, followed by a plateau at higher speeds. The close alignment of the blue dots with the red curve suggests that the ANN accurately models the thermal behavior in this range. Small deviations appear at intermediate speeds, but overall, the ANN captures the trend well near the maximum temperature.

Case (b) presents varying axial force at a constant rotational speed (n = 2250 rpm). As the axial force increases from low to high values, the temperature rises steeply initially, then levels off once the force becomes sufficiently large. The ANN predictions closely track the FEM reference curve, demonstrating good generalization across different force values. Minor discrepancies occur at mid-range forces, while the model continues providing a smooth and physically plausible temperature profile.

Overall, the ANN ID 7 network demonstrates strong agreement with reference data under both constant force and constant speed conditions. Its Tanh-Tanh-Sigmoid architecture, combined with sufficient training, enables the model to capture nonlinear thermal effects effectively.

**Figure 14:**
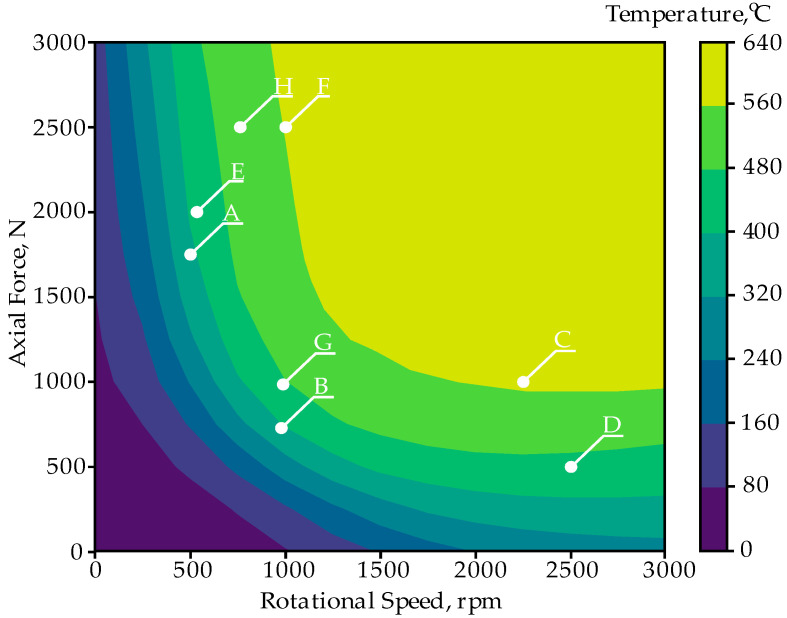
Optimization results (A–H) marked on the temperature distribution map for the ANN ID 7 architecture.

Figure 14 presents a color-coded contour map of temperature, °C predicted by the ANN ID 7 architecture, as a function of rotational speed, rpm, and axial force, N. As ID 7 is the deepest and most accurate ANN model, its predictions yield a well-defined temperature distribution. The contour map suggests that the model successfully captures nonlinear thermal effects across a broad operating range. Points A through H mark specific operating conditions identified through an optimization procedure. By examining the locations of these points on the map and their corresponding temperature values, one can assess how different combinations of rotational speed and axial force influence thermal outcomes.

The optimization points (A–H) are distributed across the diagram, covering low and high rotational speeds and forces. Each labeled point represents a trade-off between process constraints and objectives—for instance, maintaining lower temperatures while ensuring sufficient force or maximizing temperature within safe operational limits. These trade-offs depend on engineering goals, such as optimizing material properties to meet specific performance requirements.

Overall, the labeled points (A–H) on the temperature distribution map demonstrate how ANN predictions can be leveraged to optimize the RFW process, revealing different operating conditions that achieve desired temperature levels. As stated in [1], the desired peak contact surface temperature level for a Ti Grade 2/AA 5005 connector is between 380 °C and 632 °C. The solid-state welding process of dissimilar materials should be carried out below the solidus melting temperature Ts of the material with the lower Ts value. In the case of the Ti Grade 2/AA 5005 joint, it is AA 5005 aluminum alloy with Ts = 632 °C. AA 5005 is sensitive to high temperatures ranging from 200 to 250 °C and may therefore lose some of its strength. In most cases, AA 5005 does not require hot working, but if it is required, the preferred temperature range is 204 ÷ 371 °C. The annealing temperature of AA 5005 aluminum alloy is 343 °C. Based on the thermal properties of AA 5005, it was assumed that a temperature T06 = 0.6 Ts = ~380 °C at the faying surfaces allows for a good-quality weld to be obtained. In order to increase the safety margin, the RFW parameters at the temperature T08 = ~505 °C should be assumed for the faying surface.

## 5. Conclusions

This study investigated the integration of ANN and GA within a DT framework to optimize the RFW process and accurately predict temperature distributions and joint quality. Extensive numerical simulations FEM were employed to assess the impact of key parameters and the performance of various ANN architectures. As found in experimental and FEM studies, careful selection of axial force, along with rotational speed and welding time, is crucial for achieving an optimized plastic strain distribution. Optimal RFW process parameters will provide a balance between sufficient deformation and heat generation, leading to a solid joint with minimal defects. Considering the results of the conducted studies and their discussion, the conclusions drawn are described below.

### 5.1. ANN Model Performance

Different ANN architectures were evaluated, revealing that shallow networks (ID 1–2) learn quickly but exhibit unstable behavior and poor generalization. In contrast, moderate-depth networks (ID 3–4) demonstrate balanced learning and stable convergence. The deep network (ID 7), employing nonlinear Tanh-Tanh-Sigmoid activation functions and a low learning rate, achieved the highest accuracy, with predictions matching FEM data. The ANN model successfully captures the general temperature distribution, though minor inaccuracies appear in the low-force, low-speed region. High-temperature regions are well predicted, confirming the ANN’s ability to model complex thermal behaviors. The ANN model demonstrates strong predictive capabilities, with absolute and relative errors mostly below 50 °C in key operational ranges.

### 5.2. Optimization via GAs

GAs were effectively integrated with the DT to systematically explore the RFW parameter space. This approach enables the optimization of complex, multi-criteria objectives such as joint quality, defect minimization, and energy efficiency, thereby facilitating real-time adjustments and improved process performance.

### 5.3. Error Analysis and Predictive Capabilities

Comparative analyses using statistical metrics and graphical methods demonstrated that ANN predictions generally align well with FEM results—particularly in mid-range operational conditions—although discrepancies are more pronounced at extreme force and speed settings. These findings underscore the potential for further improvements in ANN training and network architecture. The temperature contour map highlights optimal operating conditions, helping engineers balance factors such as temperature, force, and speed. The A–H optimization points illustrate trade-offs in welding parameters, assisting in process optimization for different material and performance requirements over a temperature range of 380 °C to 632 °C.

Optimizing friction rotary welding parameters using AI-assisted digital twins offers many advantages, but there are several limitations to consider. First, these digital twins rely heavily on high-quality, comprehensive data from experimental measurements or finite element simulations. In friction rotary welding, process variability due to changing joint conditions, tool wear, and environmental influences can lead to data uncertainty and calibration challenges. Additionally, digital twins are typically based on simplified or idealized physical models that may not fully capture the complexities of real-world interactions, such as dynamic heat transfer and microstructural changes during welding. This means the predictions can sometimes be less reliable when extrapolating beyond the conditions covered in the training data. The optimization process can also be computationally intensive, especially when exploring a large parameter space for different welding conditions and alloy combinations. These limitations necessitate ongoing validation and refinement of the digital models with experimental results.

Ultimately, while optimization for friction rotary welding using AI-assisted digital twins is not without its limitations, it holds promise for tailoring welding parameters to achieve optimal performance, energy efficiency, and quality for a wide range of applications in automotive and aerospace. Overall, the combination of ANN-based predictive models with GA optimization within a DT framework offers a powerful, computationally efficient tool for enhancing the RFW process. The approach successfully captures complex thermal behaviors and material interactions, leading to superior joint quality and operational efficiency. Future work should focus on refining network architectures and expanding training datasets, to further improve model performance across all operating conditions.

## Figures and Tables

**Table 1:** ANN model architecture for selected hyperparameters and associated statistical metrics.

ID	Topology	Activation Functions	Learning Rate	Epoch (All/Best)	MSE *	R^2^	Training Time, s
1	2-8-1	Linear-Linear-Linear	0.1	100/61	0.157/0.175/0.157	0.6	3.94
2	2-16-1	Linear-Linear-Sigmoid	0.01	100/57	0.135/0.147/0.136	0.6848	6.55
3	2-16-1	Linear-Tanh-Sigmoid	0.01	100/100	0.125/0.137/0.130	0.7329	6.61
4	2-16-1	Linear-Tanh-Sigmoid	0.01	500/485	0.071/0.057/0.086	0.9129	33.42
5	2-16-16-1	Linear-Tanh-Tanh-Sigmoid	0.01	500/210	0.055/0.065/0.063	0.9732	89.41
6	2-16-16-1	Linear-Tanh-Tanh-Sigmoid	0.001	1000/1000	0.054/0.056/0.055	0.947	178.87
7	2-16-16-1	Linear-Tanh-Tanh-Sigmoid	0.001	5000/2038	0.045/0.045/0.045	0.9661	946.12
8	2-16-16-1	Linear-Tanh-Tanh-Sigmoid	0.0001	10,000/10,000	0.041/0.055/0.079	0.9314	1827.32

* MSE for training set/validation set/test set.

**Table 2:** Variants of the fitness functions in a genetic algorithm and their hyperparameters.

ID	F *	Constraints	Search Domain Common to All FFs	PS	NG	Hyperparameter Values Common to All FFs	ANN Record Selected by GA [n, F_A_, T]
A	f(n,T) = n + T	380 < T < 600		60	10	CR = 0.6	[500.0, 1750.0, 398]
B	f(n,T) = 1/n + T	380 < T < 600		65	15	MR = 0.2	[1000.0, 750.0, 389]
C	f(n,T) = 1/n + 1/T	380 < T < 600		70	20	SM = Tournament	[2250.0, 1000.0, 588]
D	f(F_A_,T) = F_A_ + T	400 < T < 580	500 < n < 2500	80	25	TS = 4	[2500.0, 500.0, 411]
E	f(F_A_,T) = 1/F_A_ + T	400 < T < 580	500 < F_A_ < 2500	85	30	ER = 1%	[500.0, 2000.0, 409]
F	f(F_A_,T) = 1/F_A_ + 1/T	400 < T < 580		90	35	TC = Max generations	[1000.0, 2500.0, 563]
G	f(n,F_A_) = n + F_A_	470 < T < 530		100	50	ES = Real-valued	[1000.0, 1000.0, 472]
H	f(n,F_A_) = 1/(n + F_A_)	470 < T < 530		100			[750.0, 2500.0, 510]

* Rotational Speed: n, rpm; Axial Force: F_A_, N; Temperature: T, °C. All functions are subject to minimization.

## Data Availability

The original contributions presented in this study are included in the article/Appendix A. Further inquiries can be directed to the corresponding author.

## Abbreviations

The following abbreviations are used in this manuscript:

AI	Artificial Intelligence
ANN	Artificial Neural Networks
COF	Combined Objective Function
CR	Crossover Rate
DT	Digital Twins
ER	Elitism Rate
ES	Encoding Scheme
F	Fitness Function
FEM	Finite Element Method
FF	Fitness Function
FS	Fitness Score
GA	Genetic Algorithm
HAZ	Heat-Affected Zone
ML	Machine Learning
MR	Mutation Rate
MSE	Mean Square Error
NG	Number of Generations
PS	Population Size
R^2^	R-squared
RFW	Rotary Friction Welding
RSM	Response Surface Methodology
SM	Selection Method
TC	Termination Criteria
TS	Tournament Size
UTS	Ultimate Tensile Strength

## Appendix A

The dataset from the FEM calculation. It contains 105 cases and was divided into three subsets: training set, validation set, and test set with the ratio: training (47%)/validation (36%)/test (20%). The training set was used to learn patterns. The validation set helped to tune the model and avoid overfitting. The test set was used for the final evaluation of the model performance.

**Table A1:** Dataset from the FEM calculation.

Training Set (49%)				Validation Set (34%)				Test Set (19%)			
ID	X1	X2	Z1	ID	X1	X2	Z1	ID	X1	X2	Z1
1 2 3 4 5 6 7 8 9 10 11 12 13 14 15 16 17 18 19 20 21 22 23 24 25 26 27 28 29 30 31 32 33 34 35 36 37 38 39 40 41 42 43 44 45 46 47 48 49	3000 2500 2000 1500 1000 500 0 3000 2500 2000 1500 1000 500 0 3000 2500 2000 1500 1000 500 0 3000 2500 2000 1500 1000 500 0 3000 2500 2000 1500 1000 500 0 3000 2500 2000 1500 1000 500 0 3000 2500 2000 1500 1000 500 0	0 0 0 0 0 0 0 500 500 500 500 500 500 500 1000 1000 1000 1000 1000 1000 1000 1500 1500 1500 1500 1500 1500 1500 2000 2000 2000 2000 2000 2000 2000 2500 2500 2500 2500 2500 2500 2500 3000 3000 3000 3000 3000 3000 3000	20 20 20 20 20 20 20 468 430 385 329 270 182 20 590 575 545 514 447 310 20 622 612 594 567 522 398 20 632 632 622 610 572 455 20 632 632 630 623 592 488 20 632 632 632 632 612 520 20	50 51 52 53 54 55 56 57 58 59 60 61 62 63 64 65 66 67 68 69 70 71 72 73 74 75 76 77 78 79 80 81 82 83 84 85	2750 2250 1750 1250 750 250 2750 2250 1750 1250 750 250 2750 2250 1750 1250 750 250 2750 2250 1750 1250 750 250 2750 2250 1750 1250 750 250 2750 2250 1750 1250 750 250	250 250 250 250 250 250 750 750 750 750 750 750 1250 1250 1250 1250 1250 1250 1750 1750 1750 1750 1750 1750 2250 2250 2250 2250 2250 2250 2750 2750 2750 2750 2750 2750	240 230 210 180 135 50 520 500 460 400 300 140 600 584 560 525 435 200 624 618 600 575 500 240 632 630 623 605 550 270 632 630 630 620 575 280	86 87 88 89 90 91 92 93 94 95 96 97 98 99 100 101 102 103 104 105	2250 1750 1250 750 2250 1750 1250 750 2250 1750 1250 750 2250 1750 1250 750 2250 1750 1250 750	500 500 500 500 1000 1000 1000 1000 1500 1500 1500 1500 2000 2000 2000 2000 2500 2500 2500 2500	410 360 304 235 565 530 490 395 603 585 548 485 632 620 600 540 632 630 615 570
